# Care staff’s experiences of registered nurses’ leadership close to older adults in municipal home healthcare: an interview study

**DOI:** 10.1186/s12913-026-16031-7

**Published:** 2026-11-06

**Authors:** Erica Lillsjö, Anna Willman, Lise-Lotte Jonasson, Karin Josefsson

**Affiliations:** 1 Department of Health Sciences, Faculty of Health, Science, and Technology, Karlstad University, Karlstad, 651 88 Sweden; 2 Department of Nursing Science, School of Health and Welfare, Jönköping University, Jönköping, Sweden; 3 Faculty of Nursing and Health Science, Nord University, Bodø, 8026 Norway

**Keywords:** Registered nurse, Leadership, Older adult, Care staff, Home healthcare, Municipality

## Abstract

**Background:**

Globally, there is a shift in the population distribution towards older ages. The trend is towards older adults receiving care and support at home; therefore, the need for home healthcare is expected to increase. Registered nurses lead the nursing care close to older adults in municipal home healthcare. Research indicates that registered nurses face challenges in leading care staff working close to older adults. However, less is known about care staff’s experiences of registered nurses’ leadership, which could contribute to new knowledge about registered nurses’ leadership. Therefore, this study aimed to explore care staff’s experiences of registered nurses’ leadership close to older adults in municipal home healthcare.

**Methods:**

A qualitative and explorative design with an inductive approach was used. Individual interviews were conducted with care staff (*n* = 15). Data were analysed using reflexive thematic analysis.

**Results:**

Care staff’s experiences of registered nurses’ leadership close to older adults in home healthcare were shown through three themes: Registered nurses’ leadership affect care staff’s sense of security; Registered nurses’ leadership affect care staff’s sense of being included; and Registered nurses’ closeness as leaders affects the establishment of relationships.

**Conclusions:**

Registered nurses’ leadership close to older adults in home healthcare can impact care staff’s sense of security and inclusion. Registered nurses’ closeness as leaders affects the establishment of relationships. The results of this study are relevant for registered nurses both in clinical practice and in nursing education to promote registered nurses to be nursing leaders. There is a need for the organizations to promote registered nurses’ opportunities to establish relationships with older adults and care staff to ensure quality, safe home healthcare.

**Supplementary Information:**

The online version contains supplementary material available at https://doi.org/10.1186/s12913-026-16031-7.

## Background

Globally, there is a shift in the population distribution towards older ages [1]. This means that health systems need to prepare to meet the needs of an ageing population [2]. The trend is towards older adults receiving care and support at home, enabling them to remain in their homes as long as possible [2]. In home healthcare, registered nurses (RNs) lead older adults’ nursing care [3]. In this study, this leadership is defined as RNs’ leadership close to older adults aged 65 years and older and their next of kin, and the care staff in direct nursing care. “Care staff” in this study refers to unlicensed staff, with or without education in care and social care, who provide care to older adults in their homes and perform delegated nursing care.

Previous research has shown that RNs perceive challenges in their leadership, such as communication difficulties with, and a lack of time to meet and support, care staff [4]. Next of kin have described ambiguities in RNs’ leadership of care staff performing care in the older adult’s home [5]. Also, older adults have described communication difficulties between RNs and care staff [6]. However, care staff’s own experiences are, to our knowledge, sparsely explored. Therefore, this study aimed to explore care staff’s experiences of RNs’ leadership close to older adults in municipal home healthcare.

There is an ongoing shift to care being performed in municipalities instead of hospitals [7], leading to higher demand [8] and more advanced care being performed in home healthcare [9, 10]. In Sweden, 21 regions and 290 municipalities [11] are responsible for healthcare, and most of the municipalities have responsibility for home healthcare [12]. Before home healthcare can be provided, the extent of the need for healthcare, the duration of the need, and the ability to reach a primary healthcare centre are assessed [12]. Home healthcare is governed by the Health and Medical Services Act [13], and includes nursing, medical interventions, rehabilitation, and habilitation [14]. Home healthcare is performed by licensed healthcare professionals [14] such as RNs, occupational therapists and physiotherapists [15] and care staff performing delegated nursing care [14]. RNs in home healthcare have the overall nursing responsibility and delegate nursing care to care staff [14]. Healthcare provided by physicians falls under the region’s responsibility often taking place in hospitals or primary healthcare centres [12]. Therefore, close collaboration between professionals as well between the municipalities and regions is essential for a functioning home healthcare [12].

In Sweden, the municipalities are also responsible for meeting the care and housing needs of older adults, and these services are performed mainly by care staff under the Social Services Act [16]. Therefore, care staff providing care in the older adult’s home are governed by two different laws, depending on the service they provide [12]. Care staff provide a wide range of services from basic social care to end of life care [17]. Because of increasing care needs among older adults in home healthcare, care staff have described a change in their work, with greater responsibility to perform delegated nursing care, assess the older adult’s health and report changes to the responsible RNs [17]. The high number of older adults in RNs’ responsibility makes delegating nursing care tasks, such as medication administration, insulin, catheter care and wound dressing, to care staff necessary [18]. This requires leadership from RNs [19].

Central to several definitions of “leadership” is that it implies a process and involves influencing a group of people reaching common goals [20], although there is no consensus on one definition of “leadership” [21]. Different leadership styles are described in the literature, categorized according to whether they are based on human relationships or task completion [22]. The work RNs perform in home healthcare implies leadership [23], which in this study is referred to as RNs’ leadership close to older adults, their next of kin and the care staff in direct nursing care. This definition does not include management. Previous research of RNs’ leadership close to older adults in home healthcare has shown that RNs’ leadership is complex and implies nursing responsibility, assessing the older adults’ individual needs, promoting patient safety, supporting care staff and trusting in care staff’s assessments and information concerning the older adult’s condition [3]. Managers of care staff view RNs as a support for care staff [24]. However, previous research reveals that care staff experience that they work alone in older adults’ homes and have to make their own decisions, sometimes without support [17, 25, 26].

To summarize, the need for home healthcare is expected to increase [2]. RNs face challenges in leading care staff working close to older adults [4], less is known about care staff’s experiences of RNs’ leadership. Therefore, this study aimed to explore care staff’s experiences of RNs’ leadership close to older adults in municipal home healthcare.

## Methods

### Design

A qualitative and explorative design was used. Individual interviews with Open-questions were conducted with care staff (*n* = 15) in four municipalities in Sweden. Using Open-questions makes it possible to explore participants’ experiences rather than rely on predetermined assumptions [27], as their experiences of a phenomenon contribute to knowledge and understanding [27]. Data were analysed with an inductive approach using reflexive thematic analysis according to Braun and Clarke [28], striving to explore patterns of shared meaning across the dataset. The Standards for Reporting Qualitative Research (SRQR) [29] were used for reporting purposes.

### Procedure and participants

Through a convenience sample, operations managers in four municipalities in four geographic areas in Sweden were contacted for permission to conduct interviews with care staff in their organization. The operations managers were sent an information letter outlining the study. The municipalities were located near medium-sized towns, i.e. at least 40,000 inhabitants, and rural municipalities [30]. After permission from the operations managers, the unit managers for care staff were contacted and asked to send an information letter to care staff according to the inclusion and exclusion criteria. Care staff who were interested in participating contacted the first author (EL) or their unit manager. The information letter to care staff included information about the study’s aim, and an assurance that participation was voluntary, that care staff could withdraw their participation without explanation, that data would be kept confidential, and that care staff’s identity would be protected. The letter also included contact information for the authors, allowing the care staff to ask questions. The letter explained that the interview would be conducted in a place of the care staff’s choosing, or through Zoom or via telephone. After permission from the unit managers, the first author (EL) provided oral information about the study to care staff during a workplace meeting, where possible, and to all participants before the start of the interview.

The inclusion criterion required that care staff have at least one year of work experience in municipal home healthcare for older adults. Exclusion criteria were care staff not being led by RNs in municipal home healthcare, as well as care staff working in municipal psychiatry or under the Swedish Act concerning Support and Service for Persons with Certain Functional Impairments [31].

### Data collection

The interviews were conducted in October to December 2024. EL conducted all interviews. Two test interviews were conducted to assess the logic and relevance of the questions, and the clarity of interpretation. The test interviews did not lead to any changes and were included in the study data. Four of the interviews were conducted at the care staff’s workplace, in a room allowing for private conversation. One interview was conducted thorough the video conferencing tool Zoom Workplace (Nordic Gateway for Research & Education, Kastrup, Denmark). The end-to-end encryption feature and waiting room were activated to protect data privacy. The remaining 10 interviews were held over the telephone in accordance with the care staff’s preference.

Before all the interviews were conducted, written informed consent was collected. Interviews via Zoom or telephone, were conducted from a private place to protect data privacy (EL); and the participants were likewise recommended to choose a private place. Before the interview started EL informed that care staff could withdraw their participation during the interview without explanation, that they did not have to answer the questions, and that if they had questions afterwards they could contact EL. Before the interview started, background variables, such as age and work experience in municipal home healthcare for older adults, were collected and recorded on a dictaphone. A short introduction to the subject was given, clarifying that “RNs’ leadership” referred to RNs’ leadership close to the older adult, their next of kin, and care staff, excluding management.

The interview was based on two Open-questions: “*Can you tell about your experiences of the registered nurse as a leader close to the older adult and yourself in municipal home healthcare?*” and “*Can you tell about your experiences of being led by the registered nurse in municipal home healthcare?*” Follow-up questions focusing on the study’s aim were asked if needed, such as, “*Can you tell me more?*” and “*Can you give any examples?*” Before the interview ended the care staff were asked if there was anything they wanted to add (Supplementary File 1).

The interviews lasted 11–47 min (mean 27 min; median 27 min). All interviews were recorded using a dictaphone and were transcribed verbatim using the machine transcription service Speech-to-text provided by Amberscript (Amsterdam, The Netherlands).

### Data analysis

The transcribed interviews were analysed using reflexive thematic analysis according to Braun and Clarke [28], where data were analysed in six phases exploring patterns of meaning across data. In phase one, the interviews were listened to and the transcriptions were read several times by EL, to gain familiarity with the content of the data. Notes were taken and mapping was done of reflections on potential patterns of meaning through the whole dataset. Phase two consisted of, the process of coding data where EL, systematically worked through the data, read the text closely and coded with the research aim in mind. The coding process was done in a word-document. Both semantic codes, content close to the text and latent codes, more abstracted from the text, were created. The coding process was repeated, striving for a thorough and rigorous coding process [28]. In phase three, initial themes were created by EL through a process of clustering codes together in an Excel-document, exploring patterns of shared meaning across data. The initial themes were discussed in the research group in relation to codes and data. In phase four the themes were reviewed and developed through a process of re-engagement with the data, which strengthened trustworthiness in the analysis process [28]. The themes were developed through discussions in the research group in relation to the data. In phase five the themes were refined, defined and named by identifying the essence in each theme. In phase six the analysis was completed by writing the report.

#### Reflexivity

The authors have strived to keep a reflexive approach, reflecting on how authors shape research through positionality, choices [32] and pre-understanding throughout the research process [33]. This was done by EL who kept a reflexive journal during the analysis process, and through recurring discussions in the research group throughout the analysis, to strengthen trustworthiness in the results. All authors had a pre-understanding of the context through previous research and work experience as RNs.

### Ethical considerations

Conventional research ethics principles were followed as outlined in the Declaration of Helsinki [34] and the Swedish ethics review legislation [35]. Data were pseudonymized and stored securely to prevent unauthorized access, i.e. only personnel with explicitly granted access rights. The results are presented at a group level so that no individual care staff can be identified.

## Results

The aim of this study was to explore care staff’s experiences of RNs’ leadership, defined as leadership that is close to older adults, their next of kin and the care staff in direct nursing care, excluding management. Care staff’s experienced RNs’ leadership through three themes: Registered nurses’ leadership affect care staff’s sense of security; Registered nurses’ leadership affect care staff’s sense of being included; and Registered nurses’ closeness as leaders affects the establishment of relationships (Table 1).

The themes comprise subthemes that present the care staff’s experiences of RNs’ leadership in more concrete terms. This leadership was experienced through interaction and support, with care staff serving as a link between older adults and RNs. Care staff emphasized the importance of information exchange, and that the RN included them as part of the care team. Working closely with both care staff and older adults was perceived as central in the leadership, while organizational and contextual preconditions were experienced as influencing RNs’ opportunities to remain close to daily care.

**Table 1:** Overview of care staff’s (*n* = 15) experiences of registered nurses’ leadership close to older adults in home healthcare

Subthemes	Themes
Registered nurses’ availability is essential	Registered nurses’ leadership affect care staff’s sense of security
Registered nurses’ way of interacting affects the care staff	
Registered nurses’ support promotes safe care	
Care staff act as a link between the older adults and registered nurses	Registered nurses’ leadership affect care staff’s sense of being included
Registered nurses’ information leads to a sense of wholeness	
Being a part of the team is essential	
Working close to registered nurses is essential for care staff	Registered nurses’ closeness as leaders affects the establishment of relationships
Registered nurses’ working close to the older adults is essential	
Organizational preconditions affect registered nurses’ opportunity to be close	

Fifteen care staff participated, 14 females and one male. Four worked in municipalities located near medium-sized towns and 11 worked in rural municipalities. The authors had no prior relation to the participants. The participants varied in age and work experience (Table 2).

**Table 2:** Characteristics of care staff (*n* = 15)

Characteristics		Care staff (*n* = 15)
Age, years	Mean min–max	40.7 24–62
Enrolled nurse	*n* (%)	13 (87)
Year of graduation as enrolled nurse	Median min-max	2000 1982–2019
Years worked in municipal care for older adults	Mean min–max	14.8 5–32
Years worked in municipal home healthcare for older adults	Mean min–max	8.2 1–29

### Registered nurses’ leadership affect care staff’s sense of security

RNs’ leadership affect care staff’s sense of security means that RNs as leaders affect care staff’s sense of security by being available, and through the way they interact with the care staff and support them.

#### Registered nurses’ availability is essential

The care staff experienced the importance of RNs’ availability. It was essential to them to be able to get in contact with RNs in situations, such as in acute situations, when they had noticed changes in the older adult’s health status and when questions arose during care staff’s home visits, for example regarding instructions or medication. It was important to the care staff to be able to ask in case of uncertainty and to prevent errors in the older adult’s care.

… it’s a matter of security to be able to call the registered nurse. Absolutely… you know they’re there and there is always a registered nurse 24 hours a day so that you can always call and get help … (Participant 3)

The care staff mostly contacted the RN via the phone, but also through written communication via digital media. RNs’ availability created a sense of security for the care staff and made their work run more smoothly. When RNs were not available, this could create stress, insecurity and a sense of unprofessionalism. RNs’ availability was crucial in palliative care.

… sometimes you get questions when you’re visiting someone who is in a palliative phase, that you feel a registered nurse would have responded better to than yourself … you don’t feel so professional at your job when you say that you need to call a registered nurse or ask them to come, and then it’s hard to get hold of them … (Participant 5)

RNs’ availability was experienced differently by care staff. Care staff experienced that they could always reach an RN, although not always the RN who was responsible for the older adult, such as on evenings or weekends. The care staff had also experienced difficulties getting in contact with RNs, for example when they did not have the direct number of an RN or when the RN did not answer the phone. When care staff could not reach RNs, this sometimes led to them calling an ambulance directly.

#### Registered nurses’ way of interacting affects the care staff

The care staff described how RNs’ interactions as leaders affected them and their work situation. This could be situations when RNs listened to care staff’s assessment or answered their questions, or when care staff asked for help. The RNs interaction was experienced differently by care staff and the care staff experienced that the interaction differed from RN to RN. Care staff experienced that RNs were polite, accommodating, and answered questions; they were open to discussions and suggestions and had an understanding of care staff’s work situation. Care staff had also other experiences, such as RNs being impolite and annoyed, questioning why care staff were calling, making care staff feel foolish, and asking care staff to write to them instead of calling. This made care staff feel insecure, frustrated, and a feeling of not being able to influence the situation.

… many times, it feels like, now she’s calling again and here surely comes a stupid question … and then you feel a little bit like, yes, now I’m nagging again, you don’t want to feel that … (Participant 6)

This led to the care staff avoiding to call RNs, trying to call another RN, or trying to solve the situation on their own or within the care staff group, which was time-consuming. Care staff described how they had tried to discuss this issue with both managers and RNs, without experiencing any changes.

… I know it’s not my fault, but you feel like you can’t influence that much … because I’m an enrolled nurse and I can’t call the physician, that’s how it is … (Participant 4)

#### Registered nurses’ support promotes safe care

The care staff experienced that RNs could support them as leaders, through giving advice and instructions, supporting them in different nursing situations and providing education. RNs educated care staff in new practical tasks, hygiene routines and nursing and prepared them to perform delegated nursing tasks. RNs did not push care staff into performing delegated nursing tasks, and could decide not to delegate if it appeared to them that it would not work out. Care staff experienced that RNs supported them by accompanying them in new situations. To what extent the RNs educated the care staff differed from RN to RN. RNs’ education led to a sense of security when performing new tasks in the older adult’s home.

… and if you feel insecure about a delegation or something like that … then they can come out and show and explain in a way that makes you feel more secure … or if you’re about to try something new … (Participant 1)

Care staff experienced it as positive when RNs had time set aside for education, were calm, and explained. RNs could also accompany care staff on home visits and demonstrate work tasks. RNs’ education led to a sense of development of care staff’s competence and that RNs trusted them. RNs’ education prevented errors in the older adult’s care.

… just basic things that I still carry with me so many years later, when I remove compression stockings, I hear the registered nurse who speaks and demonstrates, do it like this and you’ll see that it works out. And it still works today, I teach the same … (Participant 9)

### Registered nurses’ leadership affect care staff’s sense of being included

RNs as leaders can affect care staff’s sense of being included. Often, care staff were intermediaries between the older adults and the RNs. It was important for care staff to receive information from RNs and be included as part of the team.

#### Care staff act as a link between the older adults and registered nurses

The care staff often experienced being the ones who met the older adults and forwarded information between the older adults and the RNs, for instance concerning the older adults’ and next of kin’s questions or wishes, or changes in the older adults’ health condition as noted by the care staff. The older adult and next of kin did not always want to, as they felt, disturb RNs. Therefore, they voiced their concerns to care staff instead. Also, RNs could ask care staff to perform tasks when performing a home visit to an older adult.

… but usually, we make more visits – one or more on a daily basis. So usually, the older adult maybe tells us that he or she doesn’t feel well and we then make a decision to contact the registered nurse … (Participant 9)

Care staff experienced that RNs often performed their assessment based on care staff’s descriptions via the phone. Therefore, it was important to care staff that there should be no misunderstandings in communication with either the RN or the older adult. Care staff also experienced the importance of forwarding information to RNs, to ensure that RNs would not miss important information.

… then maybe I misunderstood what the patient said … so they might have to repeat. No, it wasn’t like that. It was like this instead. – There can be some misunderstanding and yes, so you must make an effort sometimes to understand how … what symptoms they have and so – so that you can explain further … (Participant 2)

#### Registered nurses’ information leads to a sense of wholeness

It was seen as important that RNs as leaders provide care staff with information. To perform good work, the care staff needed information. They were dependent on RNs’ mediating information, without which, they could miss important details and end up in difficult situations in meeting with the older adult and feel unprofessional. Some care staff mentioned difficulties in reading and taking part in each other’s documentation, due to accessibility to different journal systems between RNs and care staff.

… but we never get any feedback. Like, they’d call and say now we’ve done this …but rather, we have to call and ask, what have you agreed on? – … you want to know what’s been decided to be able to do a good job … (Participant 14)

The care staff had experiences of that RNs gave them oral and/or written information at reporting sessions and workplace meetings. Care staff also had experiences of lack of information from RNs, which had led to them taking more responsibility themselves, either by searching for information or by asking the older adults. The care staff experienced that RNs struggled to inform all care staff, and expressed a wish for structured information sessions, especially in palliative care.

… palliative care when the patient’s condition worsens, or something like that. And it isn’t always that they communicate to us, like, that there has been an ‘end-of-life discussion’ or that it’s been decided to discontinue all medication, and that we are to switch to injections … (Participant 5)

#### Being a part of the team is essential

According to the care staff, it was important that RNs as leaders included care staff as part of the team. The care staff had experiences of RNs and care staff not working together as a team and of RNs not involving them. Indeed, care staff could experience that they and the RNs were two separate groups who solely had contact when the RNs delegated tasks or when there was a deviation. Sometimes RNs communicated tasks via care staff managers. The care staff experienced a lack of insight into RNs’ daily work, which, they felt, affected their understanding of the RNs’ work situation.

… we are two groups … we only meet during team meetings once a month, then we go through all the older adults, and then it’s much like they have done this and we have done that, so it’s never like we’re a team … (Participant 12)

Team meetings with RNs to discuss the older adults in home healthcare were experienced as essential to get a sense of wholeness. Some care staff who were enrolled nurses, with education in care and social care, experienced that their competence, such as in taking blood samples, could be used more by RNs. This would both unburden RNs and help the enrolled nurses maintain their competence.

… we have studied, most of us working here are enrolled nurses, and then you have studied this, you have an education within these areas, and then once you’re working, you don’t get use of it because registered nurses have that responsibility … (Participant 6)

### Registered nurses’ closeness as leaders affects the establishment of relationships

RNs’ closeness as leaders affects the establishment of relationships, means that closeness of RNs as leaders was essential to the care staff and promoted establishing relationships with both the older adult and the care staff. However, organizational challenges made it difficult for RNs to be close.

#### Working close to registered nurses is essential for care staff

The care staff experienced it as easier to speak to RNs in person. Talking to RNs instead of writing to them made it easier to describe situations. It was easier to receive RNs’ help and then interventions took less time for the older adult. Care staff had experiences of working both closely with and far from RNs. Being in the same building as RNs was described as valuable. Being close contributed to an increased team spirit, and improved shared communication and information exchange between RNs and care staff. However, the care staff also experienced that even when they were located in the same building as the RNs, there was not always time to talk to or meet with RNs. They expressed the wish to have more time to meet with and talk to RNs.

… and we are in the same premises so you can consult and talk to each other at once … it doesn’t have to be so complicated when you bring it up immediately … if you suspect a urinary tract infection or something else … you can just bring it up and they can check for it at once. It becomes a faster treatment … (Participant 13)

Care staff talked about how they appreciated when RNs visited them or just called, checking in on the situation. The care staff had different experiences of how frequently RNs visited them. It was important to get to know and build trustful relationships with RNs, since it promoted a sense of security, trust in each other and a team spirit.

… we should also get to know each other better … then you would also feel more like a team … now when we only meet once a month … it feels like we don’t know where we stand with each other at all. I think it would make a huge difference if they came to us … just for a cup of coffee; it doesn’t have to be so much … (Participant 12)

#### Registered nurses’ working close to the older adults is essential

Care staff talked about how important it was that RNs as leaders worked close to the older adults. This implied that RNs should visit the older adults and follow up on their health condition, and not only be present over the phone. Older adults who had trustful relationships with RNs could find it hard to build new relationships when RNs were replaced, as happened frequently. The care staff had experiences of RNs being close to older adults making home visits, listening and involving the older adult and their next of kin. They also had experiences of nowadays, RNs paid fewer home visits and that more of their work at the older adults’ homes was placed on care staff instead, sometimes even care staff without relevant competence. This distance led to RNs not having enough knowledge about the older adults, which could lead to RNs being unable to answer care staff’s questions. Care staff and RNs rarely made home visits together, although the care staff experienced that joint visits were valuable, especially in palliative care.

… it also feels like they put more and more on us in home service. That we should do more of their work; at the same time we don’t always have the competence to do everything … (Participant 7)

The care staff experienced that older adults appreciated meeting RNs and were disappointed when the RN did not visit them. The RNs’ visits promoted their sense of security. When they rarely met RNs, care staff experienced that the older adults were a bit forgotten.

… the home visits in my experience have become fewer … we have someone who hasn’t met a registered nurse in a couple of months … and it’s a bit like, there may not be any urgent matters and then they are a bit forgotten, or how should I put it …? (Participant 6)

#### Organizational preconditions affect registered nurses’ opportunity to be close

Care staff described that organizational preconditions affect RNs’ ability to lead close. They experienced that RNs did the best they could, despite struggling in their work. They could see when RNs had a lot to do and that they were stressed. The care staff had difficulties getting to talk to RNs because RNs’ lack of time. They experienced a need for more RNs in home healthcare. RNs were often replaced and having many temporary RNs from hiring agencies led to an unstable work group. The care staff experienced RNs were responsible for the care of many older adults, and their limited time affected their ability to pay home visits and help care staff. The care staff also experienced that RNs had a lot of administrative work, and when care staff called, it implied more work for them.

… so there are many times that I feel it would have been good if there had been one or two more, so they could get some relief … – not that they become stressed and unpleasant, but they do become mostly stressed … you can tell by looking at them that they need to sit down for a moment, but there is no time … (Participant 1)

## Discussion

This study explored care staff’s experiences of RNs’ leadership close to older adults in municipal home healthcare. The results showed that RNs’ leadership affect care staff’s sense of security and their sense of being included, and that RNs’ closeness as leaders affects the establishment of relationships.

The results illuminated that RNs as leaders affect care staff’s sense of security in various ways. RNs’ availability was important to the care staff when uncertainties arose during home visits. It helped avoid errors in the older adult’s care. In previous research, care staff described that they often worked alone [17], which implies that they must deal with and solve unforeseen situations that can arise during a home visit [17, 25]. Care staff have to handle acute situations [25], as well as situations such as an older adult’s expressions of existential issues, individual needs, worries and concerns [36]. Snogren et al. [37] showed that RNs in municipal healthcare experienced dependence on the care staff reporting to them and therefore, that a functioning collaboration between care staff and RNs was crucial [37]. This is in line with the present study, where care staff experienced acting as a link between the older adult and the RN. This highlights communication and collaboration between RNs and care staff as a key factor for functioning and safe home healthcare. Therefore, municipal healthcare organizations have to facilitate the preconditions for communication and collaboration between RNs and care staff to promote safe home healthcare.

Furthermore, the results of this study revealed that care staff had different experiences, both positive and negative ones, regarding how RNs act as leaders and interact with care staff, for example when care staff called them for help or had a question. Lillsjö et al. [4] showed that RNs in home healthcare are pressured due to limited time. Moreover, RNs struggle to find the time for unforeseen situations in relation to already planned activities [38]. However, the results of this study, showing how RNs’ interaction affects care staff, are valuable and essential knowledge that can benefit RNs in clinical practice and can be used in nursing education. This knowledge can create awareness among RNs of how their leadership and encounters as leaders affect the care staff and their work situation.

This study showed the importance of RNs as leaders supporting care staff, for instance when providing education. Receiving education was experienced by the care staff to prevent errors in the older adult’s care. The development towards more advanced care being provided in home healthcare [9, 10] implies high expectations from society regarding the responsibilities of home healthcare [39]. In previous research, care staff described changes in their work, which included higher responsibility, and having to perform delegated nursing care and assess the older adult’s health [17]. The care staff in the present study experienced that RNs made fewer home visits and that more work normally done by the RN was delegated to care staff. By contrast, other research indicates a shortage of care staff with education in care [37]. Lillsjö et al. [40] reported that RNs have neither high nor low trust in care staff’s competence to assess the older adult’s condition. Therefore, RNs’ support, including education and instructions to care staff, can be seen as essential in providing safe care for older adults. This also implies a need for RNs to have time set aside to educate care staff, for example [4].

The present results revealed that through their leadership RNs can affect the care staff’s sense of being included. Receiving information and being included as part of the team was important to the care staff, enabling them to do a good job. In contrast, the results of this study also showed that care staff experienced the RNs and themselves as two separate groups that did not work together as a team. Previous research has shown that organizational structures, such as separate workgroups, can affect collaboration between professions [41]. The results of this study showed that the information care staff received was especially important in palliative care. This result aligns with previous research showing that, from RNs’ point of view, if all care staff in palliative care have the same information this is one way to create a sense of security for the patient [42]. Having information about the patient’s situation has also been described in research as making it easier to detect changes in the patient’s condition [41]. Moreover, lack of information has been identified as a risk factor for errors in home healthcare [43].

Furthermore, the results of this study showed that care staff felt dependent on RNs for information. There could be difficulties in access to each other’s documentation because the RNs and care staff used different journal systems. The Health and Social Care Inspectorate [44] in Sweden has pointed out the issue of several different documentation systems that separate healthcare and social services. This implies requirements on staff to make sure they have the information they need to avoid risking patient safety [44]. Ekman et al. [45] emphasize the importance of documentation in person-centred care. Documenting patients’ beliefs, values and involvement in care contributes to continuity of care. The results of this study showed that when care staff do not receive information from RNs, they attempt to solve this by searching for information or asking the older adult. Therefore, effective channels of information and documentation between RNs and care staff are essential, so that care staff are informed of the older adult’s situation and patient safety, as well as person-centred care can be promoted [46].

The results revealed that RNs as leaders being close promotes the establishment of relationships both with the older adult and with the care staff. This was experienced by care staff to be beneficial for the older adult’s care, as well as promoting team spirit between RNs and care staff. Care staff appreciated RNs being located in the same building, but they also appreciated RNs visiting or calling them. Therefore, “RNs being close” implies that RNs not only work in the same building, but also be present. When RNs were close, care staff experienced improved communication and information sharing.

However, the results also showed that organizational challenges, such as RNs’ time constraints and the amount of administrative work, often made it difficult for RNs to be close. Previous research has shown that organizational distance between RNs and care staff in home healthcare can result in collaboration difficulties [41]. By contrast, RNs’ availability to care staff can contribute to the establishment of relationships [47]. Jönsson et al. [48] found that the presence of RNs, when meeting with care staff in nursing homes, promotes the establishment of relationships between RNs and care staff. Therefore, the organizations need to promote close work between RNs and care staff to enable building of relationships. Further research on how to facilitate relationships between RNs and care staff may be valuable.

### Limitations

This study has some limitations. One may be that most of the interviews were held over the telephone, excluding the possibility of incorporating non-verbal communication [27]. However, telephone interviews can contribute to a feeling of privacy for a participant, enable them to participate regardless of geographic distances [27], and exclude technical interruptions which may be more prevalent when using a video conferencing tool [49]. To strengthen the trustworthiness of the study, participating care staff had to have at least one year of work experience in home healthcare for older adults, and were expected to have experience with the phenomenon [50]. The participating care staff varied in age and work experience. Of the 15 participants, one was a man, which can be seen as a limitation. However, this reflects that the majority of care staff working in care for older adults in Sweden are female [51]. A limitation to note is that RNs’ voices are not included. A limitation of the analysis may be that pre-understanding influences the reflexive approach. However, EL kept a reflexive journal throughout the analysis and held recurring discussions in the research group to strengthen trustworthiness. The descriptions of the participants and context enable the readers to assess the study’s transferability [50].

## Conclusions

RNs’ leadership close to older adults in home healthcare can impact care staff’s sense of security and inclusion. RNs’ closeness as leaders affects the establishment of relationships. The results of this study are relevant for RNs in clinical practice and nursing education as they can promote RNs to be nursing leaders, as well as for municipal healthcare organizations. There is a need for these organizations to promote RNs’ opportunities to establish relationships with both the older adults and the care staff to ensure quality and safe home healthcare.

## Supplementary Information

Below is the link to the electronic supplementary material.

Supplementary Material 1

## Data Availability

The datasets generated and analysed during the current study are not publicly available because of privacy and ethical restrictions, they are available from the corresponding author upon reasonable request.
