# Supplementary material for: Care staff’s experiences of registered nurses’ leadership close to older adults in municipal home healthcare: an interview study
Source: BMC Health Serv Res. 2026 Nov 6;26:1559. doi: 10.1186/s12913-026-16031-7 (PMC13749785; doi:10.1186/s12913-026-16031-7)
Supplement: Supplementary file 1 [file 12913_2026_16031_MOESM1_ESM.docx]

**INTERVIEW GUIDE**

**AIM**

To explore care staff’s experiences of registered nurses’ leadership close to older adults in municipal home healthcare.

**INTRODUCTION**

I would like to ask you about your experiences of registered nurses’ leadership close to older adults in municipal home healthcare, that is - leadership close to the older adult, next of kin, and you as care staff in direct nursing care. It does not include management or similar positions that are carried out far from the older adult and far from where nursing care is provided.

**QUESTIONS**

Can you tell about your experiences of the registered nurse as a leader close to the older adult and yourself in municipal home healthcare?

Can you tell about your experiences of being led by the registered nurse in municipal home healthcare?

**Follow-up questions**

In what way?

What does that mean?

Can you tell me what you mean?

Can you tell me more?

Can you give any examples?

**CLOSING**

Before we conclude the interview, is there anything additional you would like to contribute with?
